# Supplementary material for: Development of the Direct Deuteration Method for Amino Acids and Characterization of Deuterated Tryptophan
Source: Bioengineering (Basel). 2025 Sep 16;12(9):981. doi: 10.3390/bioengineering12090981 (PMC12467387; doi:10.3390/bioengineering12090981)
Supplement: Supplementary file 1 [file bioengineering-12-00981-s001.zip › bioengineering-3798553-supplementary.pdf]

bioengineering

MDPI

SUPPORTING INFORMATION

Development of the Direct Deuteration Method for Amino Acids and Characterization of Deuterated Tryptophan

Chie Shibazaki [1*], Haruki Sugiyama [2], Misaki Ueda [3], Takayuki Oku [1], Motoyasu Adachi [4], Zoë Fisher [5,6] and Kazuhiro Akutsu-Suyama [3*]

[1]J-PARC Center, Japan Atomic Energy Agency (JAEA), 2-4 Shirakata, Tokai-mura, Naka-gun, Ibaraki 319-1195, Japan
[2]Neutron Industrial Application Promotion Center, Comprehensive Research Organization for Science and Society (CROSS), 162-1 Shirakata, Tokai-mura, Naka-gun, Ibaraki 319-1106, Japan
[3]Neutron Science and Technology Center, Comprehensive Research Organization for Science and Society (CROSS), 162-1 Shirakata, Tokai-mura, Naka-gun, Ibaraki 319-1106, Japan
[4]Institute for Quantum Life Science, National Institutes for Quantum Science and Technology (QST), 4-9-1 Anagawa, Inage, Chiba 263-8555, Japan
[5]European Spallation Source ERIC, P.O. Box 176, SE-221 00 Lund, Sweden
[6]Lund Protein Production Platform, Department of Biology, Lund University, Sölvegatan 35, 22362, Lund, Sweden
* Correspondence: shibaza@post.j-parc.jp (C.S.); k_akutsu@cross.or.jp (K.A.)

1. Deuteration of Amino Acids

1.1. Tables

Table S1 Enhancement of alanine deuteration efficiency through optimization of reaction conditions.
Table S2 Effect of base and acid additives on the deuteration of leucine.
Table S3 Effect of acetic acid as an additive on the deuteration of valine and isoleucine
Table S4 Influence of ammonia as an additive and reaction temperature on the deuteration efficiency of tryptophan.
Table S5 Evaluation of deuteration conditions for tyrosine bearing a p-hydroxyphenyl side chain.
Table S6 Optimized deuteration conditions of serine and threonine with hydroxylated side chains.
Table S7 Investigation of deuteration conditions for aspartic acid, glutamic acid, asparagine, and glutamine.
Table S8 Evaluation of deuteration conditions for lysine and arginine bearing amine groups on their side chains.

1.2. Figure

Figure S1 Deuteration results of 20 standard amino acids at 200 °C for 24 hours.

2. ^{1}H and ^{2}H NMR Spectra of Products

3. X-Ray Fluorescence Spectrometry (XRF)

3.1 Figure

Figure S2. The results of the XRF analysis performed on deuterated glycine and tryptophan.

4. Fluorescence Spectra of Tryptophan After UV Degradation and Reaction with Hydrochloric Acid (HCl)

4.1. Figures

Figure S3. Time-dependent fluorescence spectra of tryptophan during the reaction with hydrochloric acid (HCl).
Figure S4. Time-dependent fluorescence spectra of tryptophan during the reaction with UV degradation.

5. X-Ray Crystallography of Tryptophan
5.1. Crystallization
5.2. X-ray crystallography
5.3. Tables
Table S9. Crystallographic data and structure refinement details.
Table S10. Bond lengths of d-Trp and h-Trp.
Table S11. Bond angles of d-Trp and h-Trp.
Table S12. Hydrogen bond lengths and angles of d-Trp and h-Trp.
Table S13. Bond lengths of d-TrpCl and h-TrpCl.
Table S14. Bond angles of d-TrpCl and h-TrpCl.
Table S15. Hydrogen bond lengths and angles of d-TrpCl and h-TrpCl.

1. Deuteration of amino acids

1.1. Tables and figures

Table S1. Enhancement of alanine deuteration efficiency through optimization of reaction conditions. Acids or bases were added in amounts equimolar to the molar amount of alanine. When NaOD was used as the additive (Entry 3), the yield was calculated based on the sodium salt form of the product.

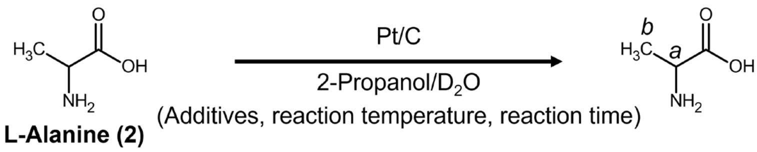

Substrate No.	Entry	Additives	Reaction temp. (℃)	Reaction time (h)	Yield (%)	Deuterium incorporation[1] (%) a	b	Mean D ratio[2]
2	1	-	200	24	93.6	97.0	27.0	44.0
	2	CH_3COOD	200	24	97.4	90.0	8.5	16.0
	3	NaOD	200	24	150.0	92.8	27.3	43.7
	4	NH_3	200	24	88.9	95.4	45.4	57.7
	5	NH_3	230	24	54.2	97.8	54.9	65.6
	6	NH_3	230	152	30.9	95.4	76.1	80.9

[1] Determined by ^{1}H-NMR spectroscopy.
[2] "Mean D ratio" refers to the mean deuteration ratio (%).

Table S2. Effect of base (Entry 2) and acid (Entry 3) additives on the deuteration of leucine. The reaction was carried out at 200 °C for 24 hours, with approximately 11 mmol of additives used.

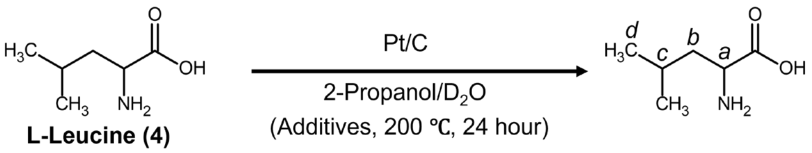

Substrate No.	Entry	Additives	Yield (%)	Deuterium incorporation[1] (%) a	b	c	d	Mean D ratio[2]
4	1	-	80.4	96.5	20.4	20.4	12.1	23.0
	2	NH_3	75.5	95.7	9.3	9.3	1.5	13.2
	3	CH_3COOH	83.9	98.5	39.6	39.6	22.6	35.3

[1] Determined by ^{1}H-NMR spectroscopy.
[2] "Mean D ratio" refers to the mean deuteration ratio (%).

Table S3. Effect of acetic acid as an additive on the deuteration of valine and isoleucine. The reaction was carried out at 200 °C for 24 hours using a Pt/C catalyst, with approximately 11 mmol of additives used.

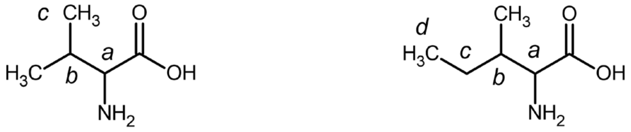

L-Valine (3) **L-Isolucine (5)**

Sub-strate No.	Entry	Additives	Yield (%)	Deuterium incorporation[1] (%)				
				a	*b*	*c*	*d*	Mean D ratio[2]
3	1	-	90.5	96.5	8.4	0.2	-	13.3
	2	CH_3COOH	105.0	99.2	75.6	74.5	-	77.8
5	3	-	61.4	97.5	18.3	2.2	0.0	12.0
	4	CH_3COOH	73.8	95.7	6.5	1.4	0.0	7.8

[1] Determined by ^{1}H-NMR spectroscopy.
[2] "Mean D ratio" refers to the mean deuteration ratio (%).

Table S4. Influence of ammonia as an additive and reaction temperature on the deuteration efficiency of tryptophan. The symbol "minus" indicates that the compound decomposed and thus the deuteration ratio could not be measured. The reactions were conducted at 200, 170, and 140 °C for 24 h (Entries 1–3, respectively), using approximately 11 mmol of additives.

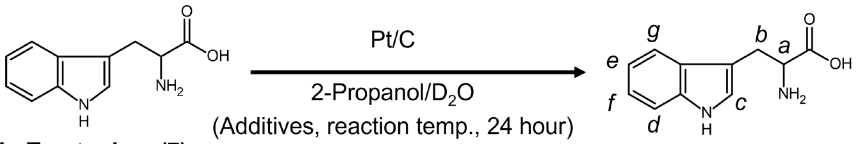

L- Tryptophan (7)

Sub-strate No.	Entry	Additives	Reaction temp. (℃)	Yield (%)	Deuterium incorporation[1] (%)							
					a	*b*	*c*	*d*	*e*	*f*	*g*	Mean D ratio[2]
7	1	-	200	-	-	-	-	-	-	-	-	-
	2	NH_3	170	85.7	93.3	43.5	95.9	32.3	91.5	89.8	95.1	73.1
	3	NH_3	140	84.3	32.6	10.3	95.2	6.8	61.2	61.2	61.2	42.4

[1] Determined by ^{1}H-NMR spectroscopy.
[2] "Mean D ratio" refers to the mean deuteration ratio (%).

Table S5. Evaluation of deuteration conditions for tyrosine bearing a p-hydroxyphenyl side chain. The "–" symbol in Entry 1 indicates that the compound decomposed and the deuteration ratio could not be measured, while the "–" symbol in Entry 4 indicates that the compound did not react and the deuteration ratio could not be measured.

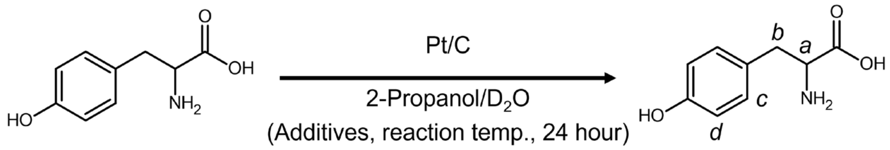

L- Tyrosine (10)

Sub-strate No.	Entry	Additives[3]	Reaction temp. (℃)	Reaction time (h)	Yield (%)	Deuterium incorporation[1] (%) *a*	*b*	*c*	*d*	Mean D ratio[2]
10	1	-	200	24	-	-	-	-	-	-
	2	CH_3COOH	170	24	45.1	96.8	17.3	18.4	95.6	51.4
	3	NH_3	170	24	86.1	90.9	2.8	7.6	89.8	41.6
	4	-	140	24	-	-	-	-	-	-

[1] Determined by ^{1}H-NMR spectroscopy.
[2] "Mean D ratio" refers to the mean deuteration ratio (%)
[3] Due to its low solubility, two equivalents of the additive were used.

Table S6. Optimized deuteration condition of serine and threonine with hydroxylated side chains. The "minus" symbol indicates that the compound was decomposed and thus the deuteration ratio could not be measured. In Entry 6, ruthenium on carbon (Ru/C) was used instead of platinum on carbon (Pt/C) as the catalyst, with the molar amount of catalytic metal adjusted to be equivalent.

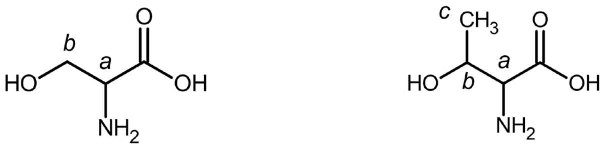

L- Serine (8) **L-Threonine (9)**

Sub-strate No.	Entry	Catalyst	Reaction temp. (℃)	Reaction time (h)	Yield (%)	Deuterium incorporation[1] (%) *a*	*b*	*c*	Mean D ratio[2]
8	1	Pt/C	200	24	-	-	-	N/A	-
	2	Pt/C	170	24	-	-	-	N/A	-
	3	Pt/C	100	24	91.9	16.7	3.2	N/A	7.7
9	4	Pt/C	200	24	-	-	-	-	-
	5	Pt/C	100	24	-	-	-	-	-
	6	Ru/C	100	24	92.8	6.3	6.3	1.2	3.3

[1] Determined by ^{1}H-NMR spectroscopy.
[2] "Mean D ratio" refers to the mean deuteration ratio (%).

Table S7. Investigation of deuteration conditions for aspartic acid, glutamic acid, asparagine, and glutamine**.** Pt/C was used as the catalyst. The "minus" symbol indicates that the compound was decomposed and therefore the deuteration ratio could not be measured.

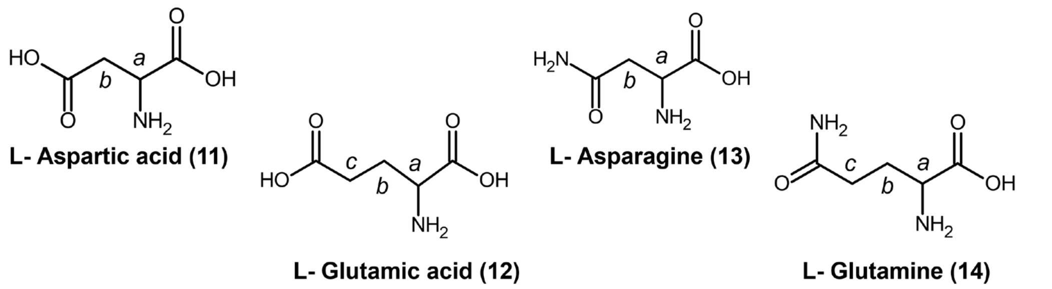

Sub-strate No.	Entry	Additives	Reaction temp. (℃)	Reaction time (h)	Yield (%)	Deuterium incorporation[1] (%) *a*	*b*	*c*	Mean D ratio[2]
11	1	-	200	24	-	-	-	N/A	-
12	2	-	200	24	-	-	-	-	-
13	3	-	200	24	-	-	-	N/A	-
14	4	-	200	24	-	-	-	-	-

[1] Determined by ^{1}H-NMR spectroscopy.
[2] "Mean D ratio" refers to the mean deuteration ratio (%).

Table S8. Evaluation of deuteration conditions for lysine and arginine bearing amine groups on their side chains. Pt/C was used as the catalyst. The "minus" symbol indicates that the compound was decomposed and therefore the deuteration ratio could not be measured.

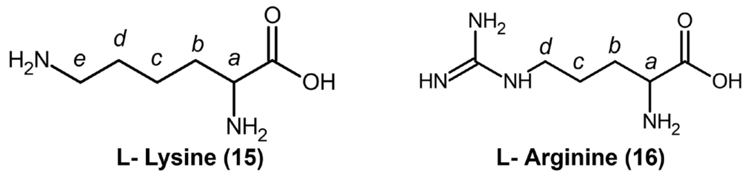

Sub-strate No.	Entry	Additives	Reaction temp. (℃)	Reaction time (h)	Yield (%)	Deuterium incorporation[1] (%) *a*	*b*	*c*	*d*	*e*	Mean D ratio[2]
15	1	-	200	24	-	-	-	-	-	-	-
	2	-	100	24	-	-	-	-	-	-	-
16	3	-	200	24	-	-	-	-	-	N/A	-
	4	-	100	24	93.7	23.6	7.8	7.8	23.6	N/A	14.6

[1] Determined by ^{1}H-NMR spectroscopy.
[2] "Mean D ratio" refers to the mean deuteration ratio (%).

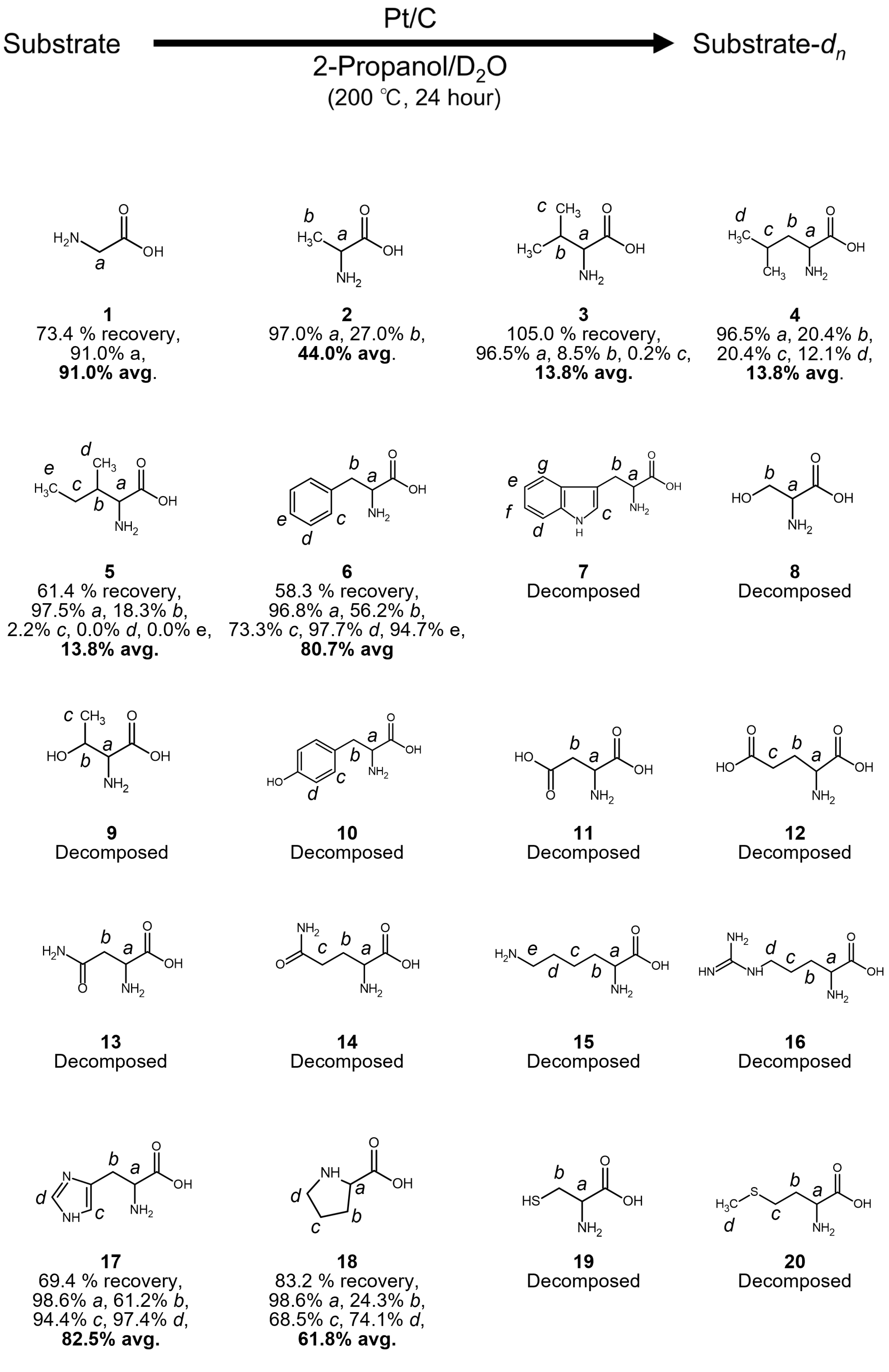

Figure S1. Deuteration results of 20 standard amino acids at 200 °C for 24 hours. Each amino acid (1 g) was reacted with Pt/C (3 wt% Pt, 0.40 g, 0.06 mmol) in a mixed solvent of 2-propanol (4 mL) and

D_2O (40 mL). The numbers shown below each molecular structure correspond to the substrate numbers in Fig. 1. Italic letters (*a-f*) indicate the positions of carbon atoms bearing hydrogen atoms, and the percentages represent the corresponding deuteration levels. Bold values indicate the mean degree of deuteration (%), as determined by NMR spectroscopy.

2. ^{1}H and ^{2}H NMR Spectra of Products

The deuteration levels of amino acids were evaluated from the integral value of the 1H NMR signals arising from the deuterated amino acids and the DMSO internal standard. Each deuterated amino acid (about 10 mg) and DMSO (about 10 mg), which were precisely measured and placed in the same vial together for each tare, were dissolved in D_2O (0.75 μL). Since aspartic acid, glutamic acid, asparagine, glutamine, tyrosine, and lysine contained large amounts of impurities in their crude products, we think that these deuteration reactions were unsuccessful.

Glycine (blank test, deuteration level is 5.5%):

^{1}H NMR (400 MHz, D_2O, DMSO) δ (ppm) 3.54 (s, 1.89 H).

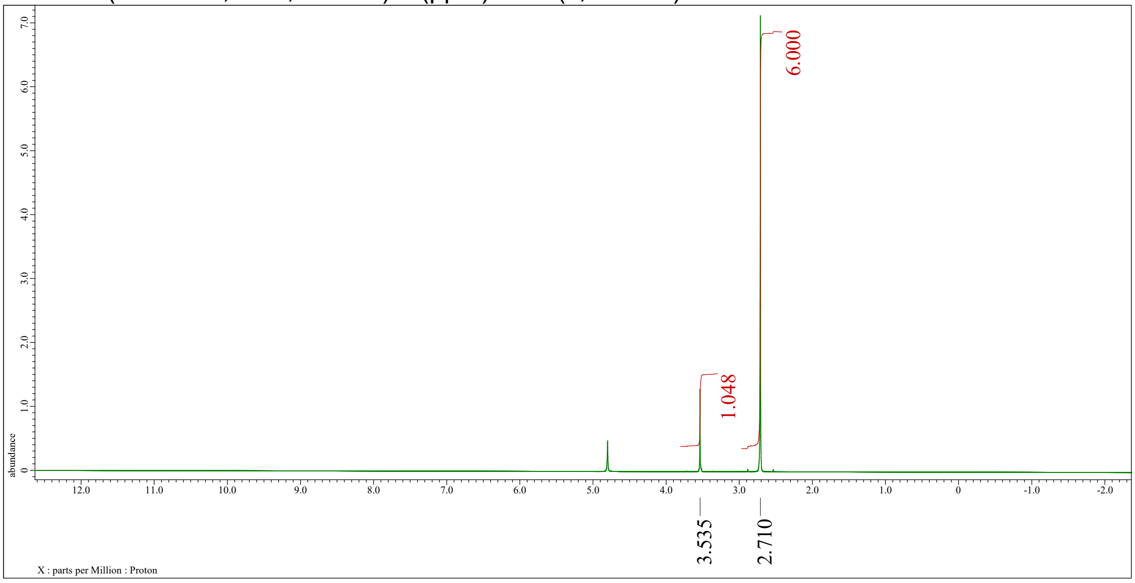

^{2}H NMR (61.4 MHz, D_2O) δ (ppm) 3.48 (br).

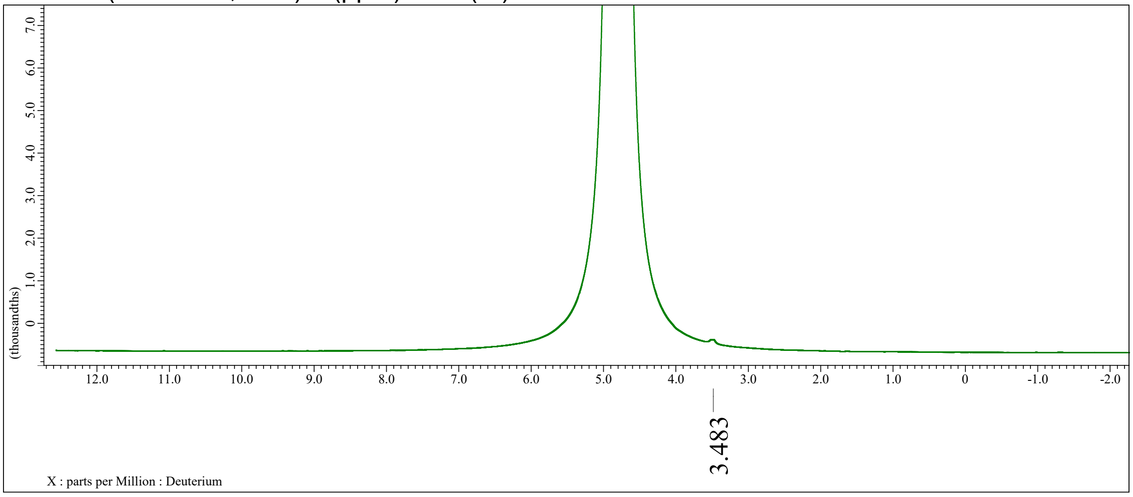

Glycine (deuteration level is 91%):

^{1}H NMR (400 MHz, D_2O, DMSO) δ (ppm) 3.49–3.51 (m, 0.09 H).

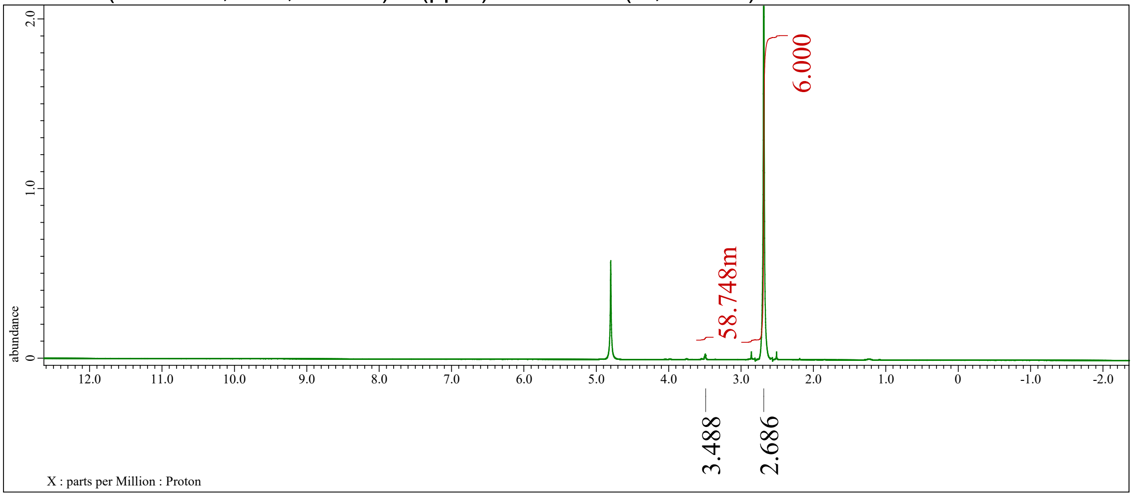

^{2}H NMR (61.4 MHz, D_2O) δ (ppm) 3.47 (br).

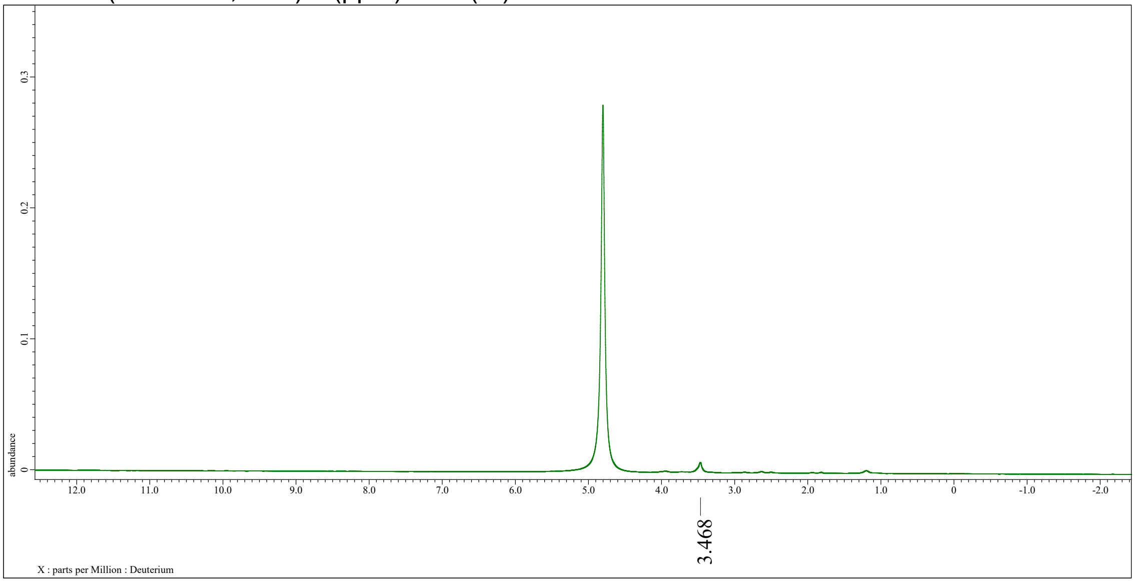

Alanine (deuteration level is 80.9%):

^{1}H NMR (400 MHz, D_2O, DMSO) δ (ppm) 1.43 (s, 0.73 H), 3.72 (s, 0.03 H).

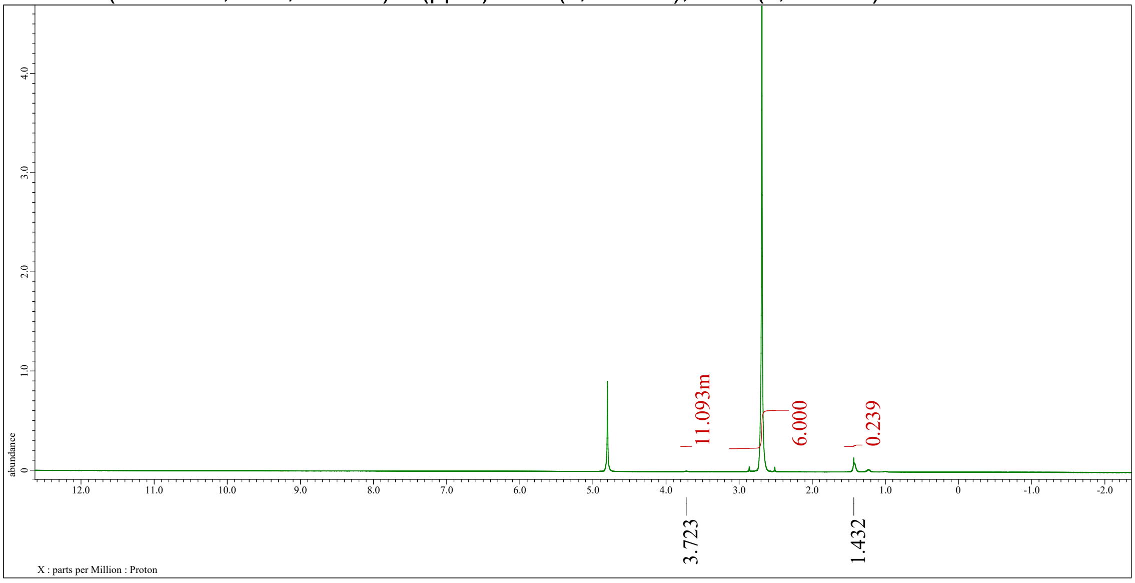

^{2}H NMR (61.4 MHz, D_2O) δ (ppm) 1.39 (br), 3.68 (br).

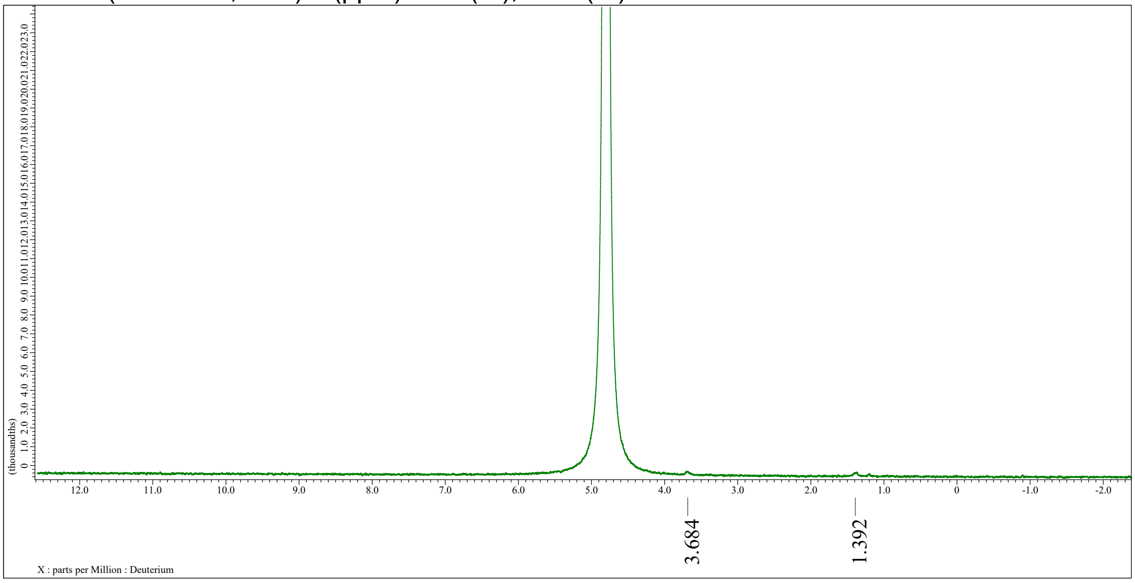

Valine (deuteration level is 77.7%):

^{1}H NMR (400 MHz, D_2O, DMSO) δ (ppm) 0.94–1.01 (m, 1.53 H), 2.15–2.29 (m, 0.24 H), 3.56 (s, 0.01 H).

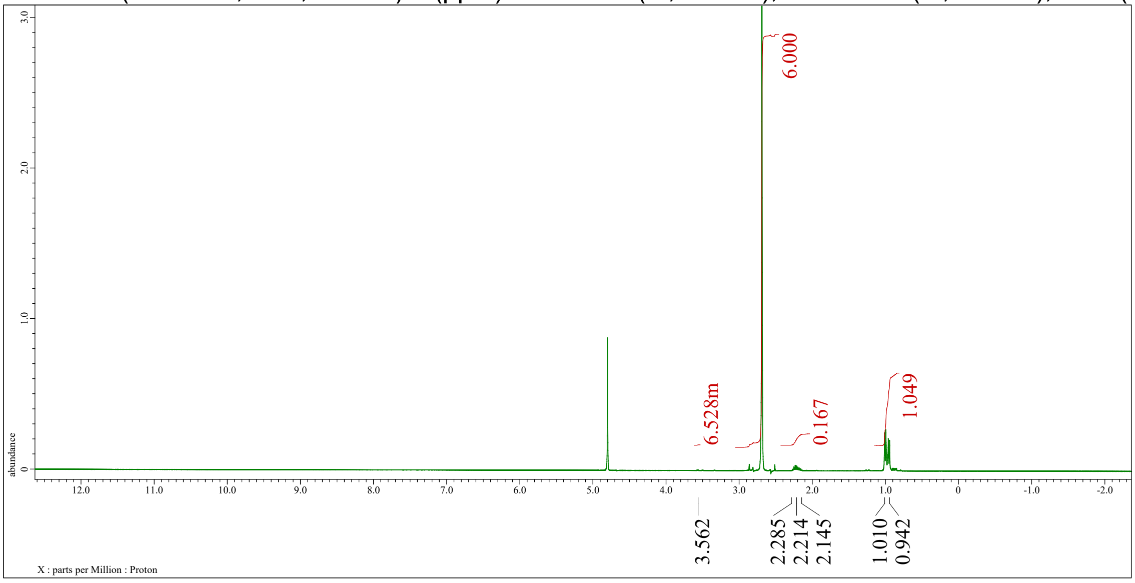

^{2}H NMR (61.4 MHz, D_2O) δ (ppm)1.19 (br), 1.90 (m, 0.24 H), 3.53 (s, 0.01 H).

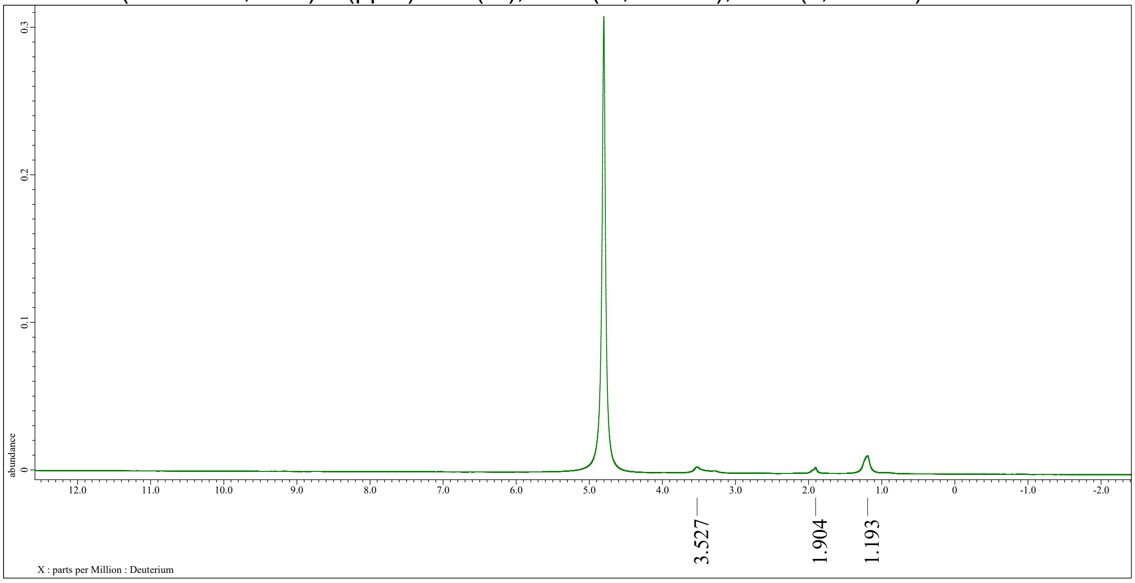

Leucine (deuteration level is 35.3%):

^{1}H NMR (400 MHz, D_2O, DMSO) *δ* (ppm) 0.83–0.93 (m, 4.65 H), 1.59–1.75 (m, 1.81 H), 3.81 (s, 0.02 H).

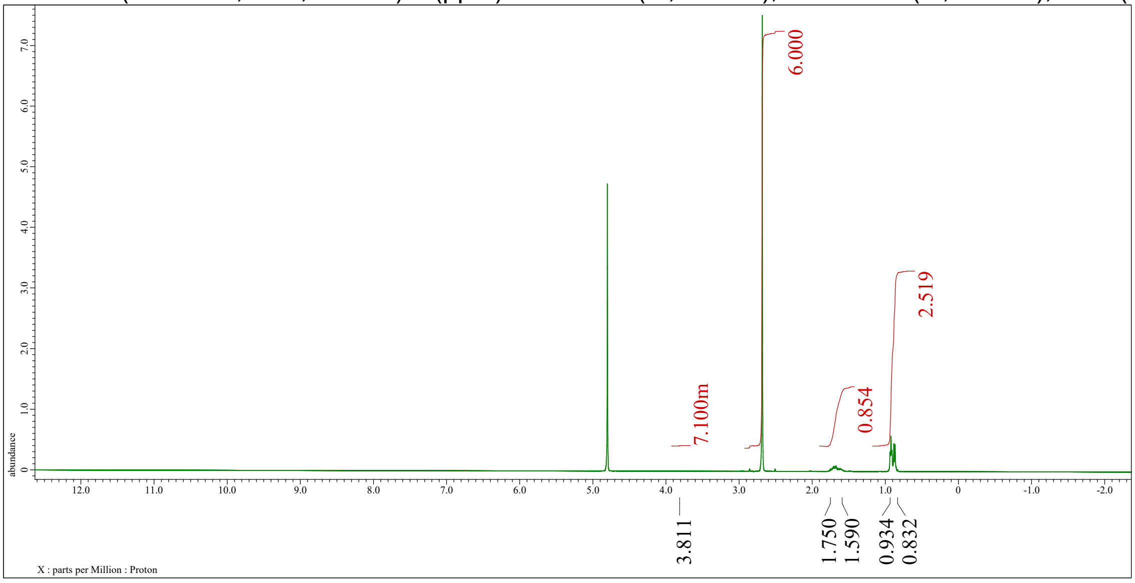

^{2}H NMR (61.4 MHz, D_2O) δ (ppm) 1.68 (br), 3.66 (br).

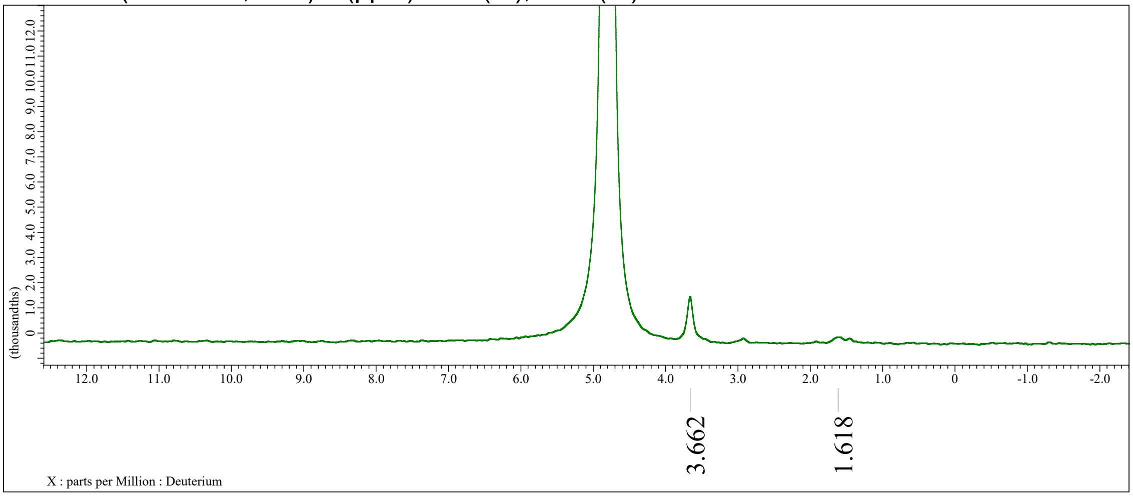

<u>Isoleucine (deuteration level is 7.8%):</u>

^{1}H NMR (400 MHz, D_2O, DMSO) *δ* (ppm) 0.90–1.00 (m, 6.0 H), 1.18–1.48 (m, 1.97 H), 1.91–2.07 (m, 0.94 H), 3.64–3.72 (m, 0.04 H).

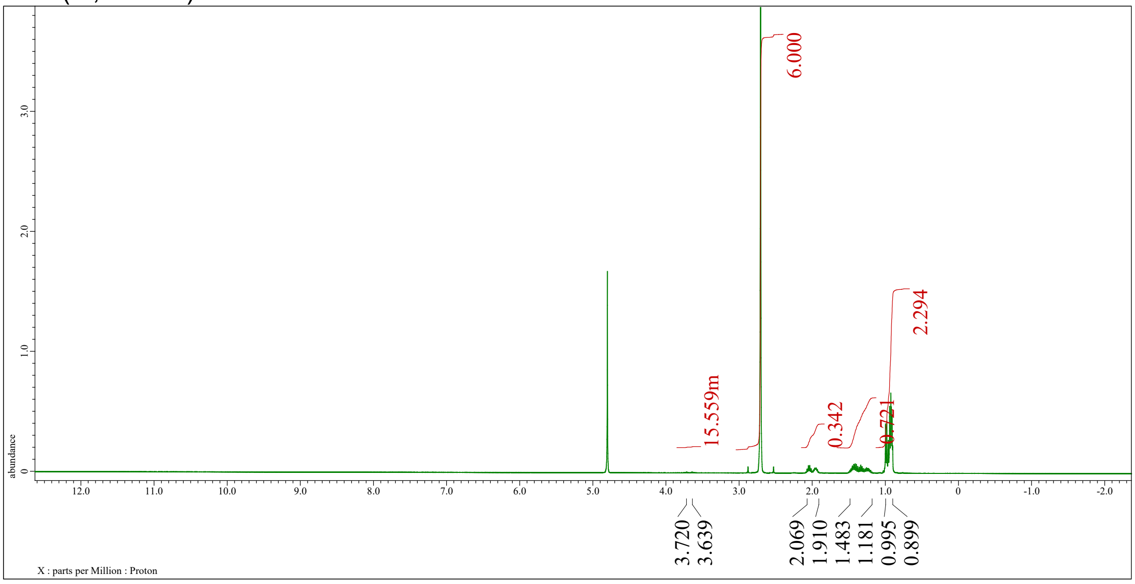

^{2}H NMR (61.4 MHz, D_2O) δ (ppm) 3.66 (br).

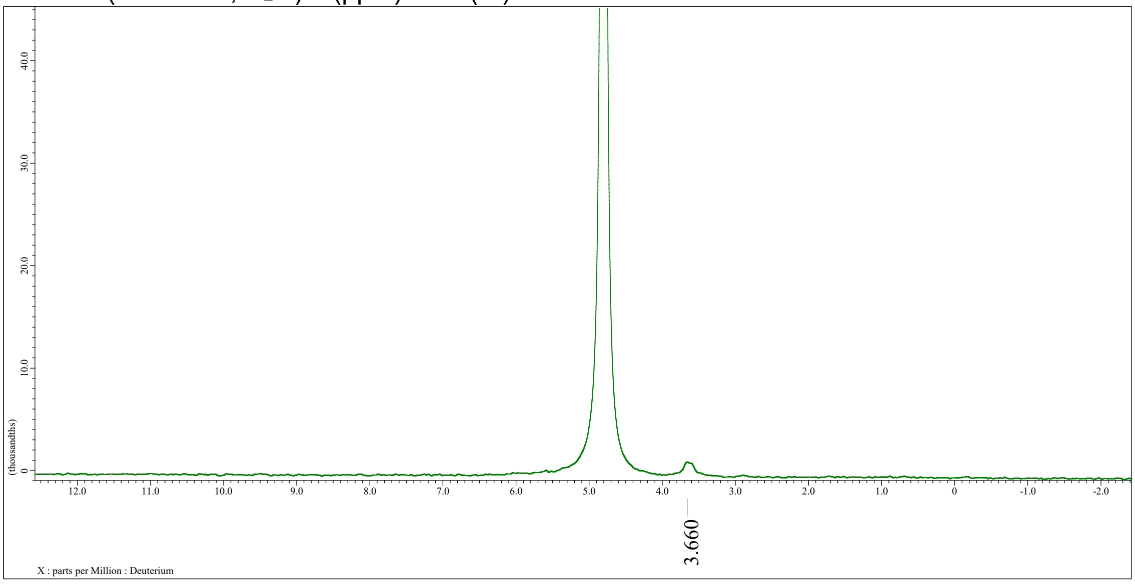

Phenylalanine (deuteration level is 80.7%):

^{1}H NMR (400 MHz, D_2O, DMSO) *δ* (ppm) 3.06–3.27 (m, 0.88 H), 3.94–3.95 (m, 0.03 H), 7.27–7.40 (m, 0.63 H).

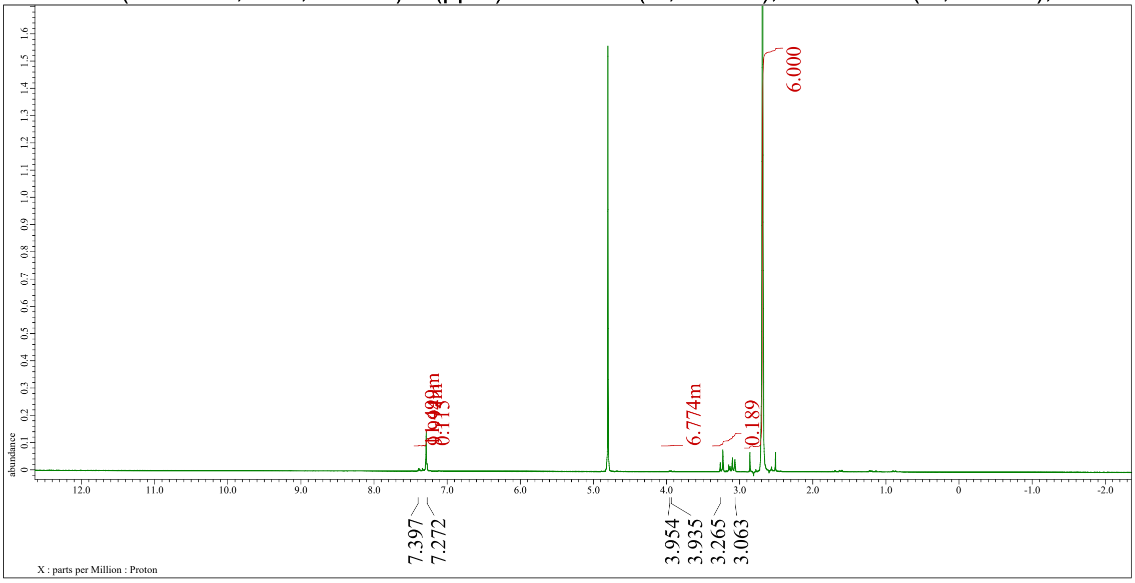

^{2}H NMR (61.4 MHz, D_2O) δ (ppm) 3.04–3.20 (br), 3.92 (br), 7.40 (br).

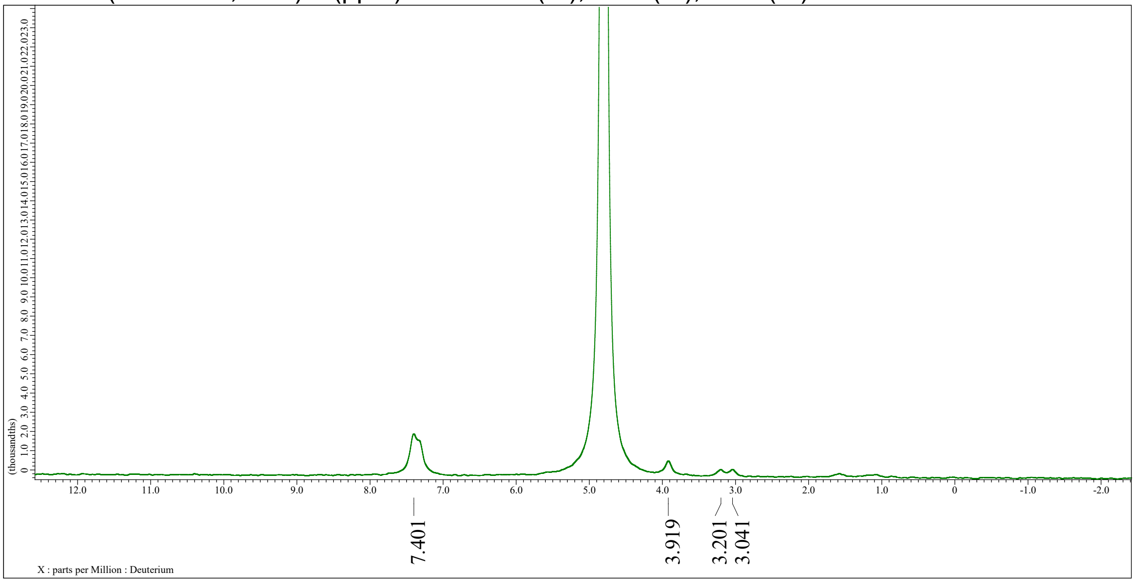

<u>Tryptophan (deuteration level is 73.1%):</u>

^{1}H NMR (400 MHz, D_2O, DMSO) *δ* (ppm) 3.37–3.50 (m, 0.74 H), 4.33 (brs, 0.01 H), 7.14–7.29 (m, 0.22 H), 7.49 (s, 0.05 H), 7.65 (s, 0.68 H).

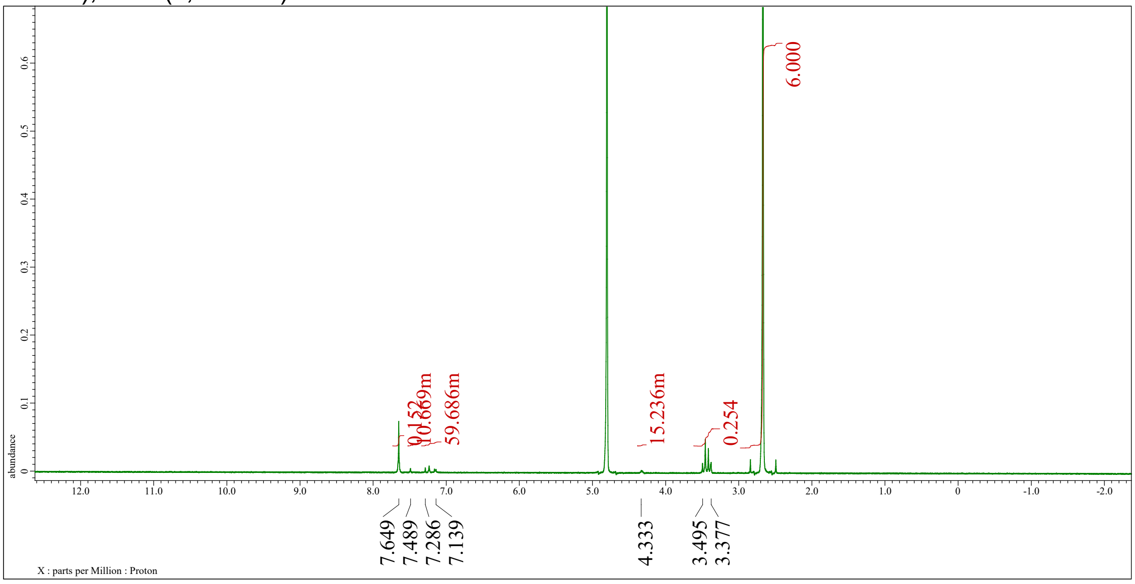

^{2}H NMR (61.4 MHz, D_2O) δ (ppm) 3.39 (br), 4.27 (br), 7.19–7.52 (br).

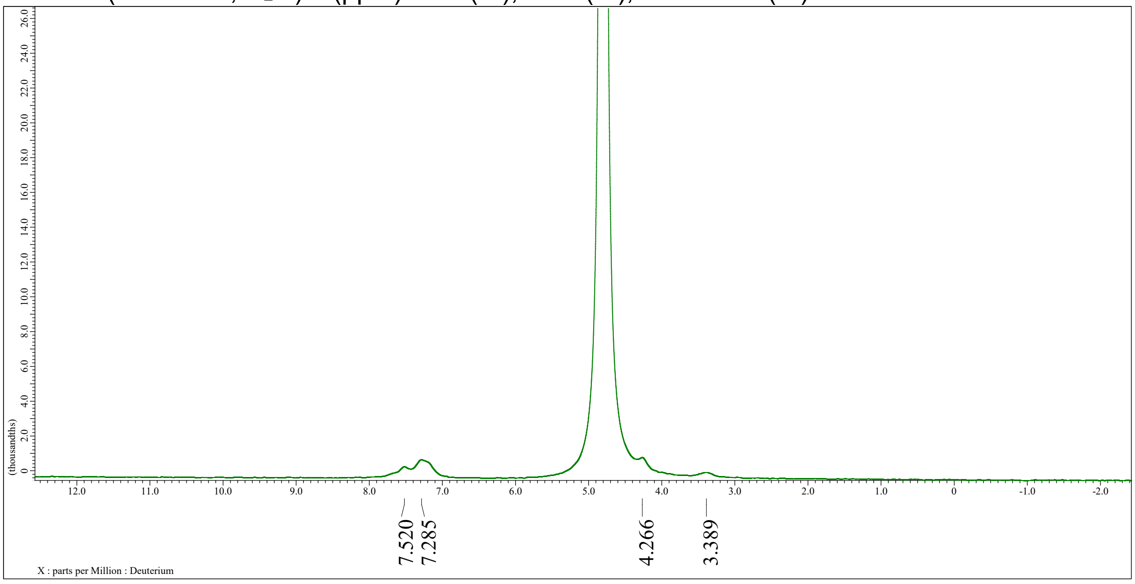

Serine (deuteration level is 7.7%):

^{1}H NMR (400 MHz, D_2O, DMSO) δ (ppm) 3.79–3.81 (t, 0.83 H), 3.88–3.97 (m, 1.94 H)

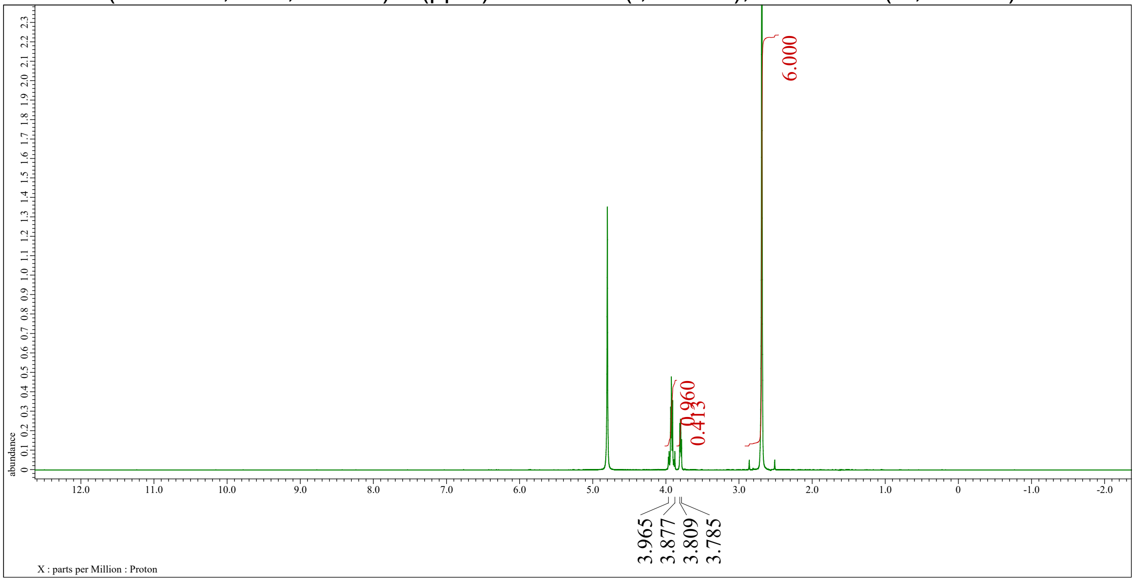

^{2}H NMR (61.4 MHz, D_2O) δ (ppm) 3.68 (br).

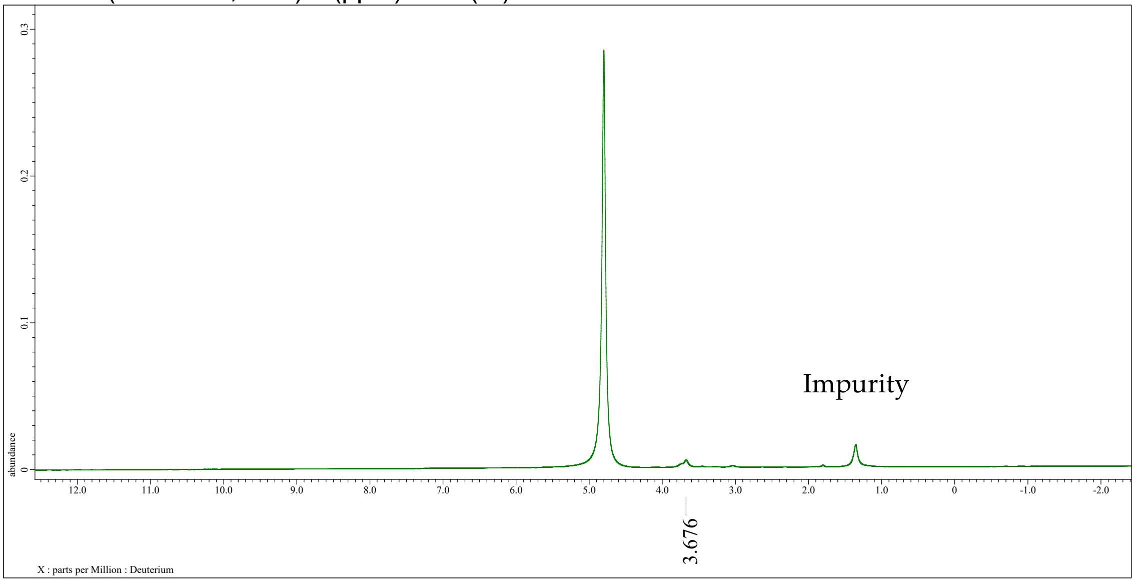

Threonine (deuteration level is 3.3%):

^{1}H NMR (400 MHz, D_2O, DMSO) *δ* (ppm) 1.28–1.29 (d, 2.96 H), 3.53–3.55 (d, 0.94 H), 4.18–4.24 (m, 0.94 H).

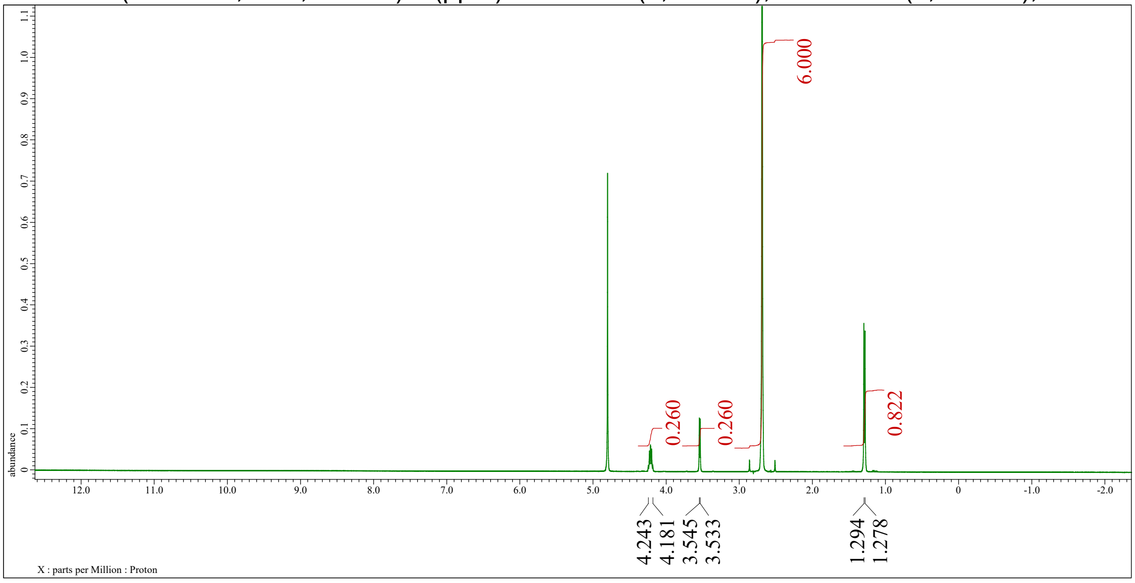

^{2}H NMR (61.4 MHz, D_2O) δ (ppm) 3.51 (br).

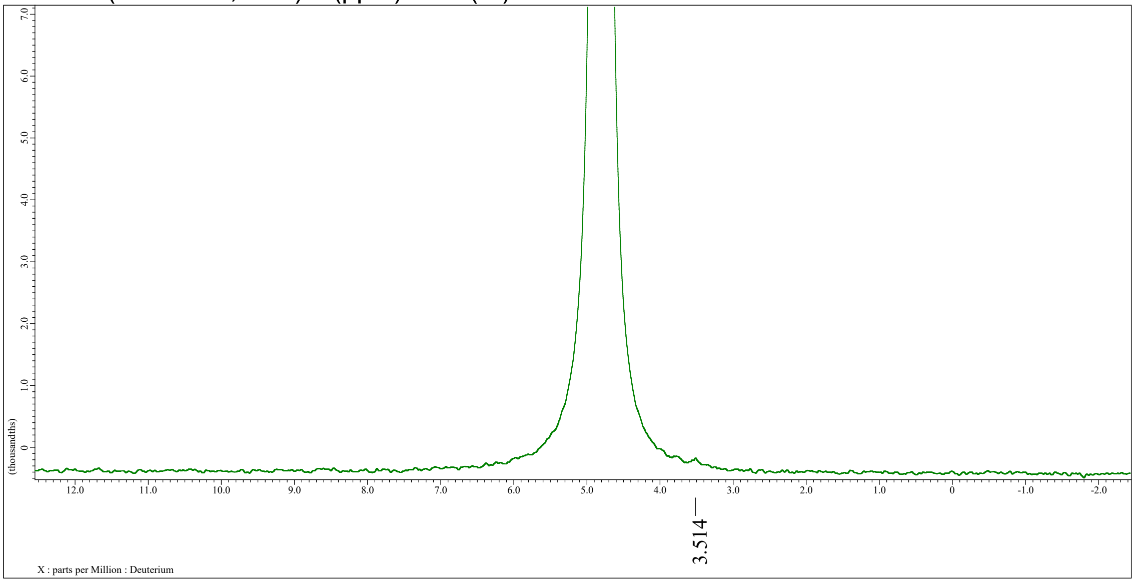

Arginine (deuteration level is 14.6%):

^{1}H NMR (400 MHz, D_2O, DMSO) δ (ppm) 1.54–1.64 (m, 3.69 H), 3.15–3.27 (m, 2.29 H).

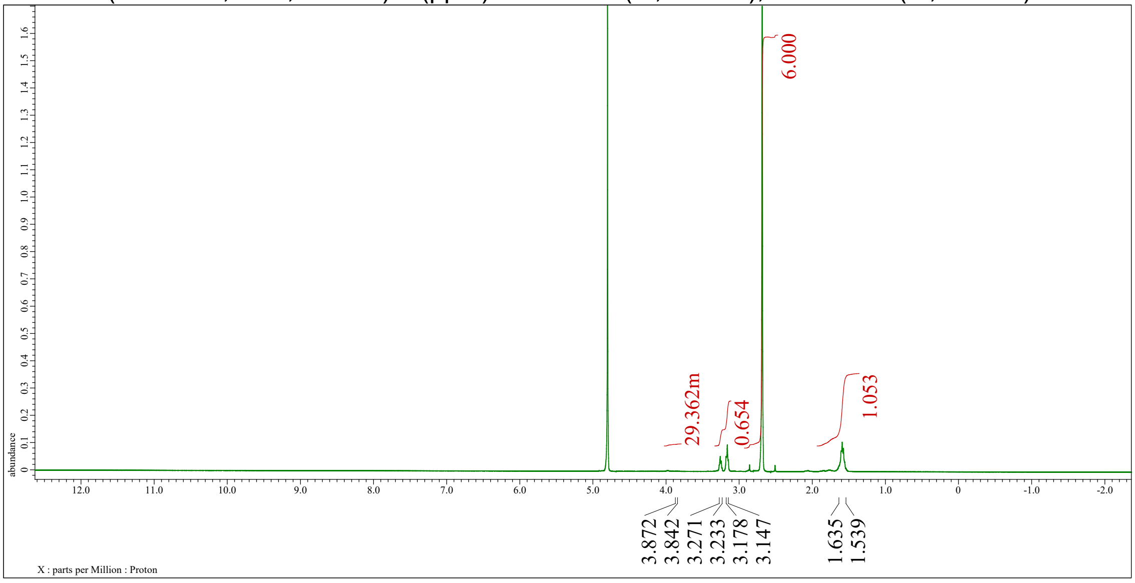

^{2}H NMR (61.4 MHz, D_2O) δ (ppm) 3.26 (br).

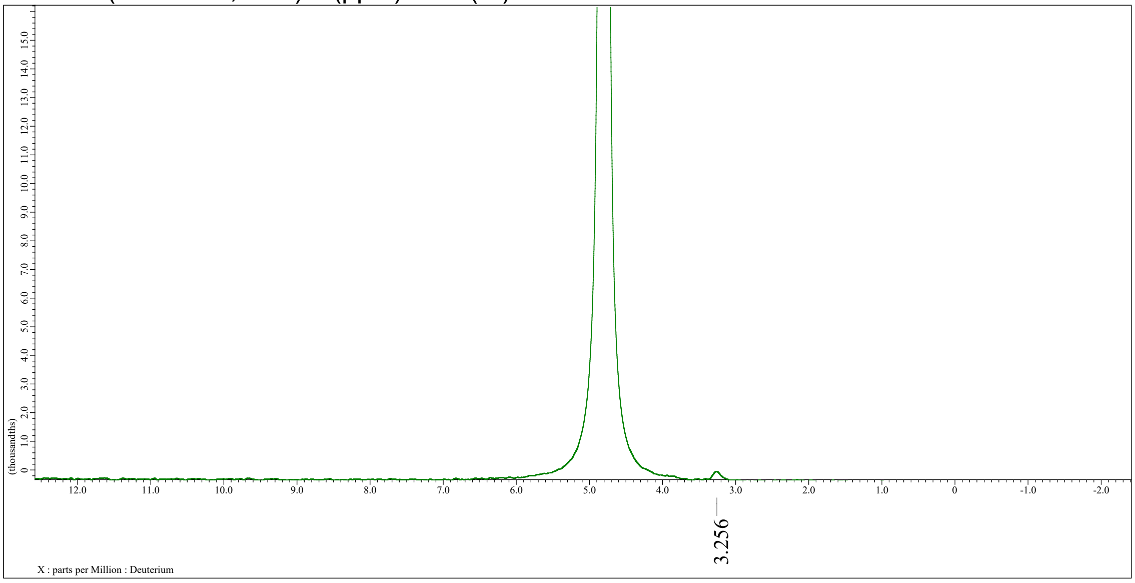

Histidine (deuteration level is 84.3%):

^{1}H NMR (400 MHz, D_2O, DMSO) δ (ppm) 3.03–3.18 (m, 0.78 H), 3.91–3.94 (m, 0.01 H), 6.99 (s, 0.03 H), 7.69 (s, 0.06 H).

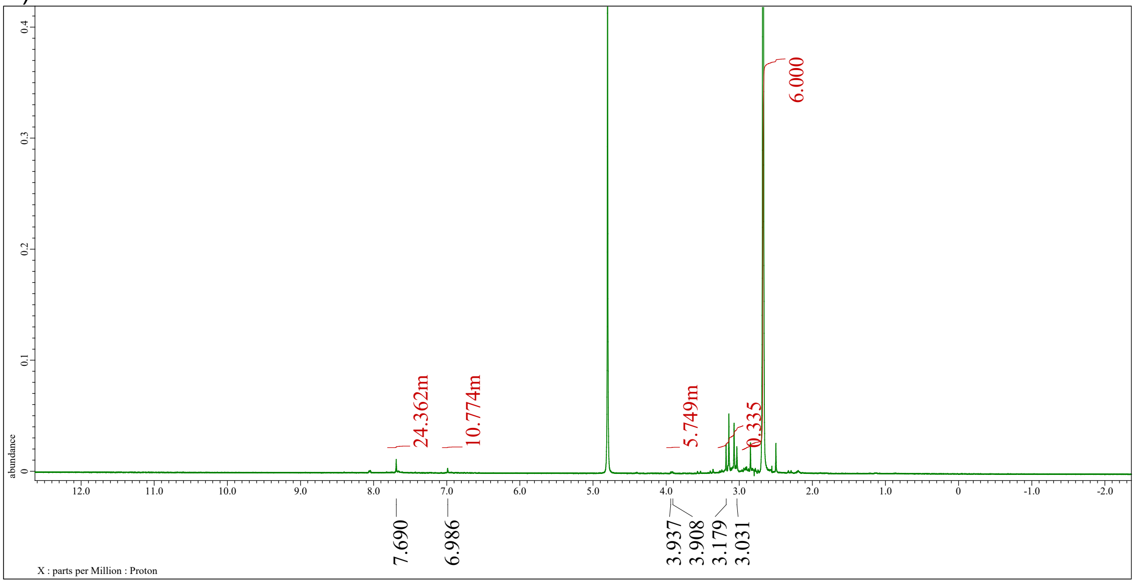

^{2}H NMR (61.4 MHz, D_2O) δ (ppm) 3.90 (br), 7.02 (br), 7.73 (br).

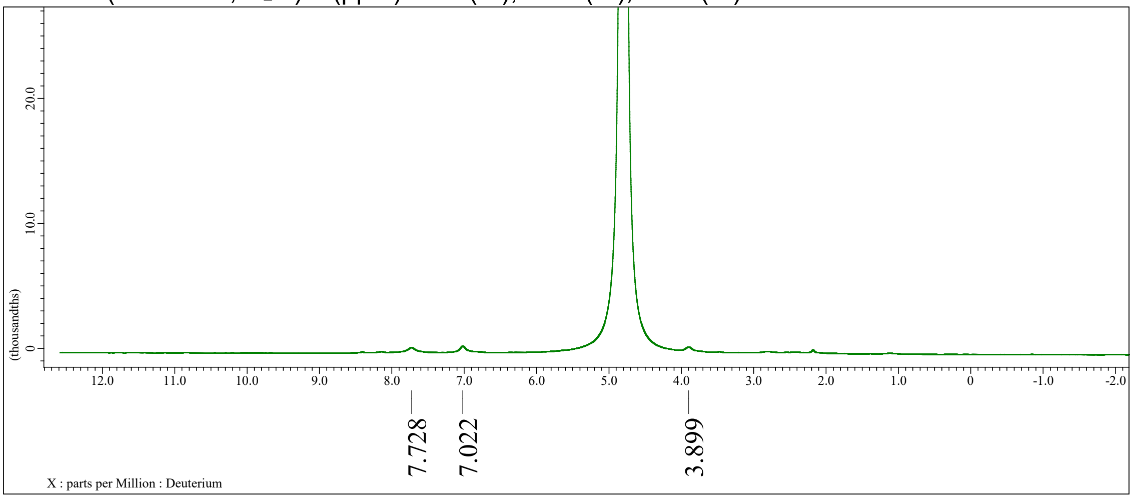

Proline (deuteration level is 61.8%):

^{1}H NMR (400 MHz, D_2O, DMSO) δ (ppm) 1.97–2.12 (m, 1.39 H), 2.32–2.40 (m, 0.76 H), 3.29–3.45 (m, 0.52 H), 4.10–4.13 (m, 0.01 H).

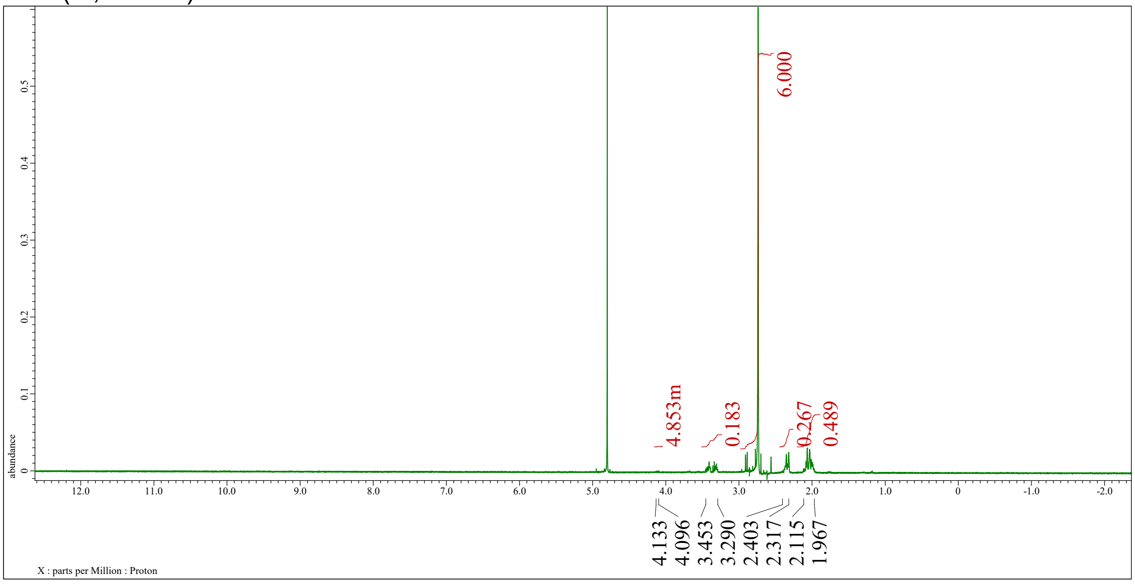

^{2}H NMR (61.4 MHz, D_2O) δ (ppm) 1.91 (br), 2.33 (br), 3.24–3.33 (br), 4.05 (br).

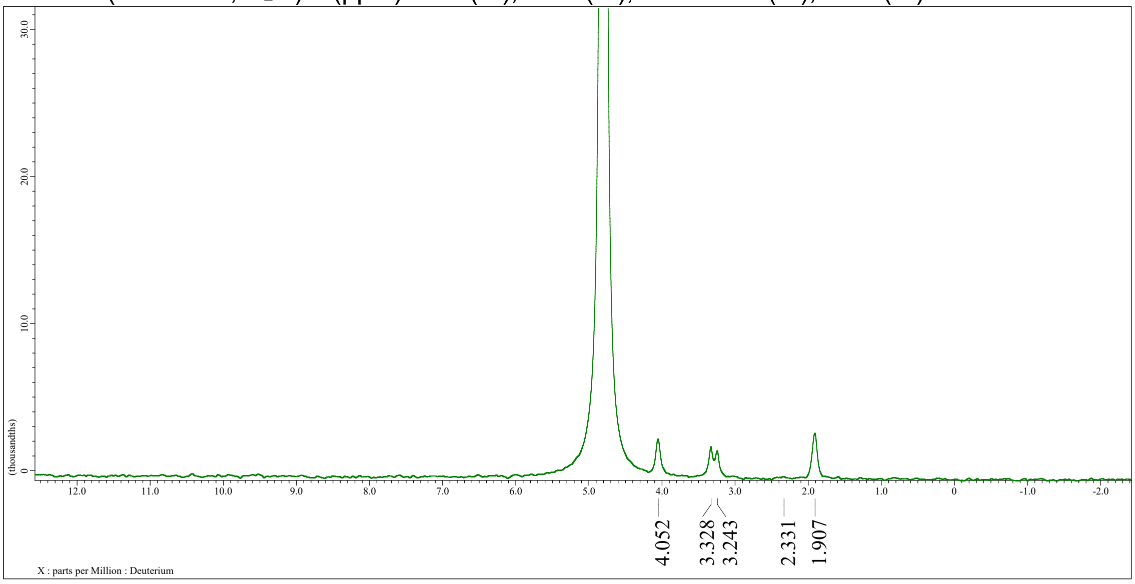

Tyrosine (deuteration level is 61.8%):

^{1}H NMR (400 MHz, D_2O, DMSO) *δ* (ppm) 2.97–3.17 (m, 1.65 H), 4.18–4.22 (m, 0.03 H), 6.77–6.79 (m, 0.09 H), 7.01–7.08 (m, 1.63 H).

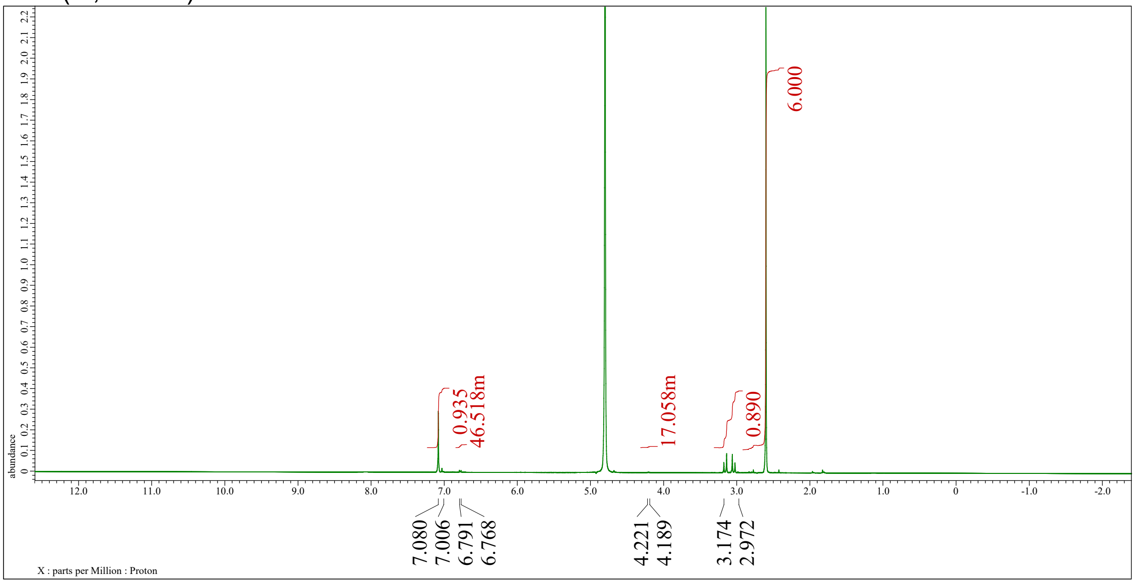

^{2}H NMR (61.4 MHz, D_2O) δ (ppm) 4.20 (br), 6.86 (br).

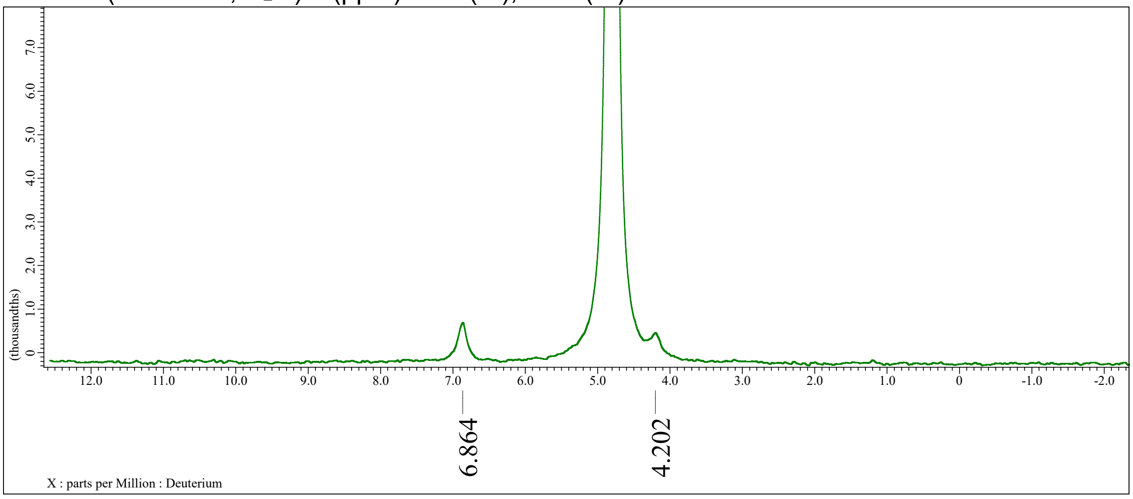

3. X-Ray Fluorescence Spectrometry (XRF)

Element analysis was performed by an X-ray fluorescence spectrometer (Shimadzu Lab Center XRF-1700, Japan) equipped with a Rh X-ray tube and 4 kW generator. The X-ray source was operated at 50 kV and 0.5–0.7 mA, and the total measurement time was 300 sec.

The Pt/C catalysts used in the deuteration experiments were used for XRF analysis in their original state. Since it was difficult to perform XRF analysis of all deuterated amino acids, the deuterated glycine (91%) and tryptophan (73.1%) were used for XRF analysis to examine whether metal ions were present in the deuterated amino acids. The measurement samples were placed in plastic cups and then installed in a vacuum chamber. All measurements were performed in vacuum to avoid X-rays being scattered by air.

3.1. Figure

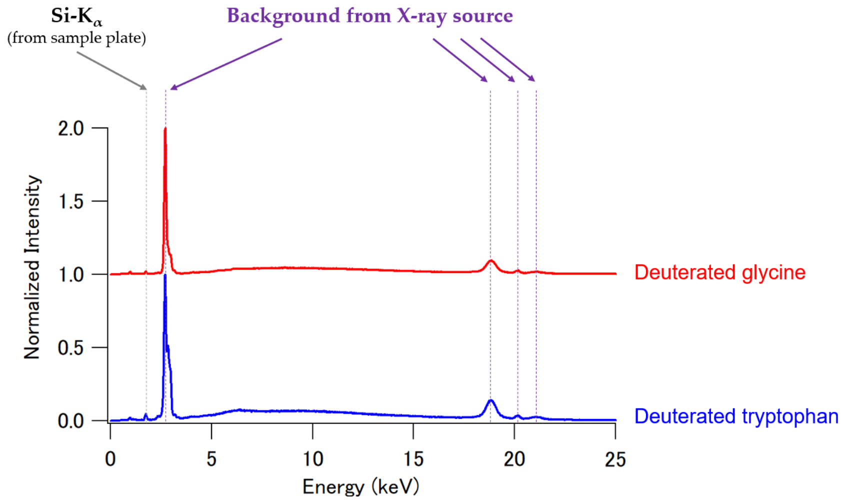

Figure S2. The results of the XRF analysis performed on deuterated glycine and tryptophan.

4. Fluorescence Spectra Of Tryptophan After UV Degradation And Reaction With Hydrochloric Acid (HCl).

4.1. Figures

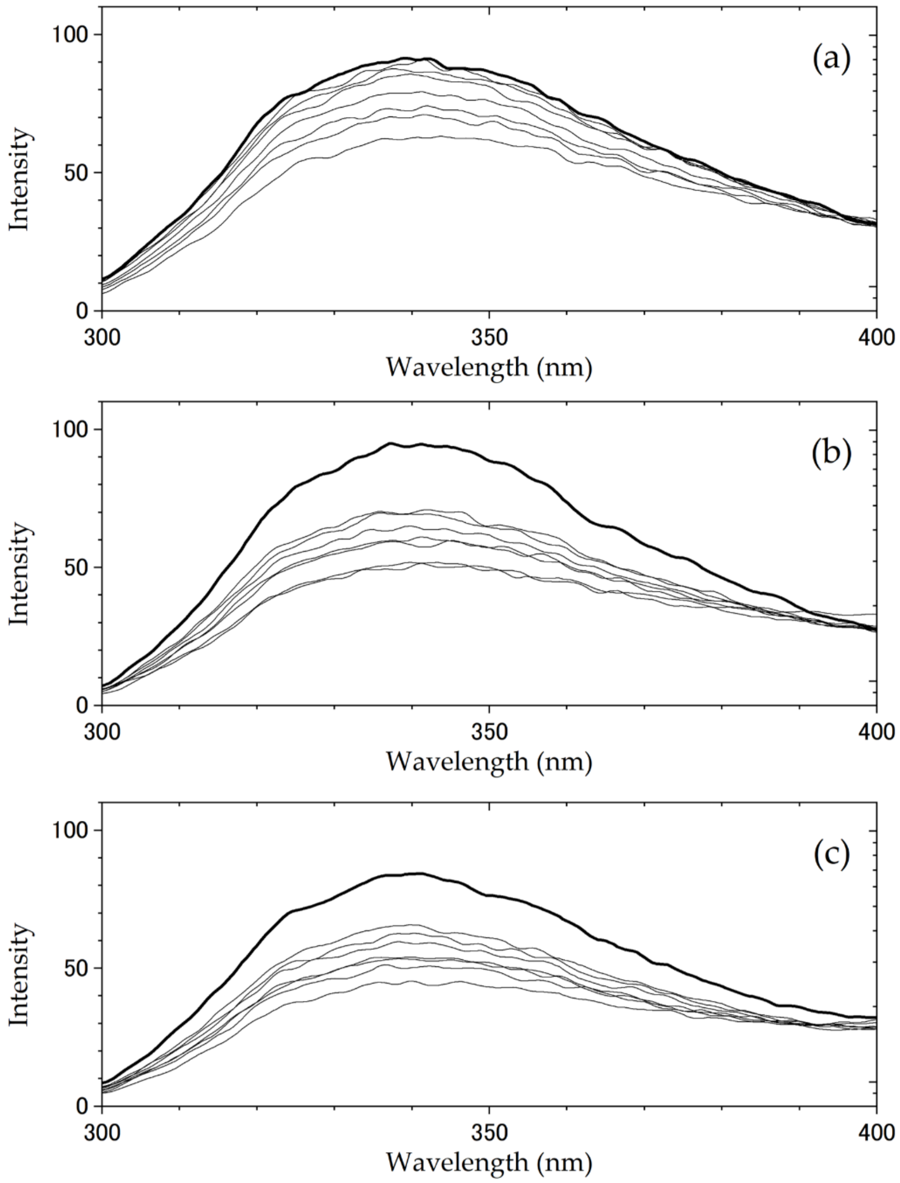

Figure S3. Time-dependent fluorescence spectra of tryptophan during the reaction with hydrochloric acid (HCl). In each panel, the bold line represents the fluorescence spectrum at the start of the reaction (time zero). (a) h-Trp spectra were recorded at 0 min (bold line), 10 min, 30 min, 60 min, 120 min, 240 min, 360 min, 720 min, and 1440 min. The fluorescence intensity around 340 nm gradually decreased over time. (b) d_{40}-Trp and (c) d_{70}-Trp spectra were recorded at 0 min (bold line), 30 min, 60 min, 120 min, 240 min, 360 min, 720 min, and 1440 min. A similar time-dependent decrease in fluorescence intensity around 340 nm was observed in both cases.

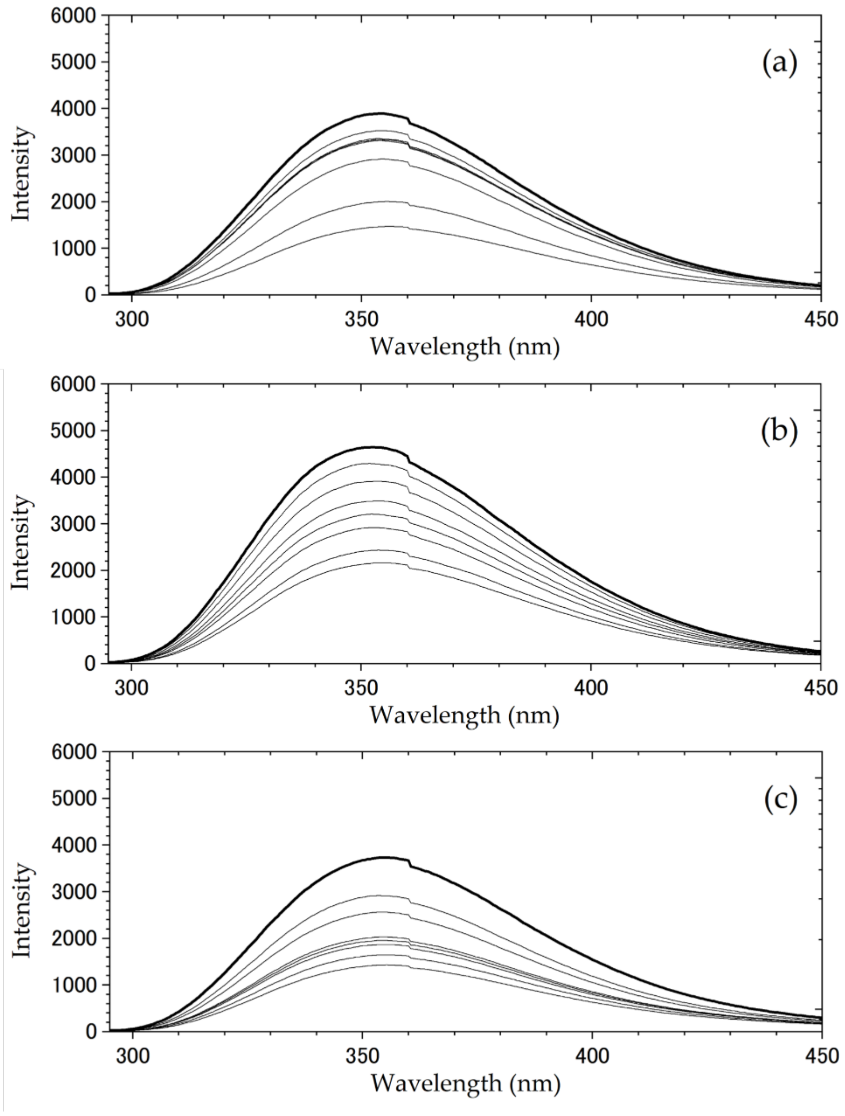

Figure S4. Time-dependent fluorescence spectra of tryptophan during UV-induced degradation. In each panel, the bold line represents the fluorescence spectrum at the start of the reaction (time zero). (a) h-Trp, measured at 0 min (bold line), 5 min, 10 min, 20 min, 30 min, 60 min, 120 min, 180 min, 240 min, 360 min, and 480 min. (b) d_{40}-Trp, measured at 0 min (bold line), 5 min, 10 min, 20 min, 30 min, 60 min, 120 min, 180 min, 240 min, 360 min, 720 min, and 900 min. (c) d_{70}-Trp, measured at 0 min (bold line), 5 min, 10 min, 20 min, 30 min, 60 min, 120 min, 180 min, 240 min, 360 min, 540 min, 720 min, and 900 min. In all cases, the fluorescence intensity around 350 nm decreased progressively over time.

5. X-ray Crystallography of Tryptophan

5.1. Crystallization

Deuterated tryptophan (d-Trp)
Deuterated DL-tryptophan (10 mg) was dissolved in formic acid 200 μL. Plate-like colorless crystals were obtained by the vapor diffusion method with ethyl acetate as the less soluble solution after few days.

Tryptophan (h-Trp)
Tryptophan crystals were obtained as following previous work (Hübschle 2004). DL-tryptophan (10 mg) was dissolved in a mixture of 2-propanol and formic acid (1:1) 2 mL. Plate-like colorless crystals were obtained via slow evaporation under ambient conditions after one week.

Deuterated tryptophan chloride (d-TrpCl)
Deuterated DL-tryptophan (14.2 mg) was dissolved in 0.15M DCl/D_2O solution 530 μL. Rod-like colorless crystals were obtained via slow evaporation under ambient conditions after one week.

Tryptophan chloride (h-TrpCl)
DL-tryptophan (20.6 mg) was dissolved in 0.33M HCl 420 μL Plate-like colorless crystals were obtained via slow evaporation under ambient conditions after one week.

5.2. X-Ray Crystallography

A suitable crystal was mounted on a glass capillary and transferred to the rugged two-axis goniometer of a RIGAKU diffractometer with equipped with mirror monochromated Mo-Kα radiation (λ = 0.71073 Å) and a HyPix-6000HEIC detector. Cell parameters were determined and refined, and raw frame data were integrated using CrysAlisPro 1.171.43.98a (Rigaku OD, 2023). Since d-Trp crystals were obtained as twin, the diffraction peaks were integrated referring to an established twin matrix, and the reflection data were merged as HKLF 5 format for crystal structure refinement. The structures were solved by dual methods using SHELXT and refined by full-matrix least-squares techniques against F2 with SHELXL- 2019/3 using the Olex2 software package. The deuteration ratio (H:D ratio) for all hydrogen atoms of tryptophan was referenced against the result of NMR and recrystallization conditions. For d-Trp and d-TrpCl, the H:D ratios for the hydrogen atoms of the methylene sp3-carbon (C6) and indole sp2-carbon (C12) were fixed to be 50:50 and 67:33, respectively. Moreover, the hydrogen atoms of the amino group were considered to be exchanged with light hydrogens in d-Trp because the crystals were obtained by recrystallization from non-deuterated formic acid. All non-hydrogen atoms were refined with anisotropic displacement parameters. For both h-TrpCl, and d-TrpCl, the hydrogen atoms were located from the difference Fourier map and refined with isotropic displacement parameters. The absolute structures were determined by refinement of the Flack parameters. For h-Trp, the hydrogen atoms were located from the difference Fourier map and refined, with their Uiso values constrained to 1.2 times those of all other carbon and nitrogen atoms. For d-Trp, the hydrogen atoms of an amino group (H5A, H5B, H5C), an indole sp2-nitrogen (D9), and sp2-carbon (D13) were were refined isotropically on calculated positions using a riding model, with their Uiso values constrained to 1.2 times those of the nitrogen atoms. The N-H, N-D, and C-D bond lengths were refined to be excessively short without any constraints. Other hydrogen atoms were located from the difference Fourier map and refined, with their Uiso values constrained to 1.2 times those of all other carbon and nitrogen atoms[48]. Details of the crystal data and a summary of the intensity data collection parameters are listed in Table S8.

5.3. Tables

Table S9. Crystallographic data and structure refinement details.

	h-Trp	d-Trp	h-TrpCl	d-TrpCl
formula	$C_{11}H_{12}N_2O_2$	$C_{11}H_{4.67}D_{7.33}N_2O_2$	$C_{11}H_{13}ClN_2O_2$	$C_{11}H_{1.67}ClD_{11.34}N_2O_2$
fw	204.23	211.6	240.68	252.09
T (K)	100	100	100	100
λ (Å)	0.71073	0.71073	0.71073	0.71073
cryst syst	monoclinic	monoclinic	monoclinic	monoclinic
space group	$P2_1/c$	$P2_1/c$	$P2_1$	$P2_1$
a (Å)	18.9284(10)	19.020(2)	7.4677(3)	7.4726(5)
b (Å)	5.7557(3)	5.7570(6)	5.2873(2)	5.2779(4)
c (Å)	9.3049(5)	9.2681(12)	14.5602(7)	14.5735(11)
α (deg)	90	90	90	90
β (deg)	101.688(5)	101.617(13)	98.890(4)	98.869(7)
γ (deg)	90	90	90	90
V (Å^3)	992.71(9)	994.0(2)	567.99(4)	567.90(7)
Z	4	4	2	2
D_{calc} (g·cm^{-3})	1.366	1.411	1.407	1.474
ε (mm^{-1})	0.096	0.096	0.323	0.323
F(000)	432	432	252	252
cryst size (mm)	0.20 × 0.20 × 0.02	0.20 × 0.05 × 0.02	0.15 × 0.05 × 0.02	0.15 × 0.05 × 0.02
2θ range (deg)	4.394 to 61.01	6.554 to 61.016	5.522 to 61.004	5.518 to 61.004
reflns collected	19788	7636	11905	11955
indep reflns/R_{int}	3028 / 0.0275	3689 / -	3411 / 0.0260	3451/0.0387
params	172	159	197	197
GOF on F^2	1.081	1.112	1.054	1.067
R_1, wR_2 [I>2σ(I)]	0.0441, 0.1083	0.0649, 0.1680	0.0245, 0.0605	0.0287, 0.0652
R_1, wR_2 (all data)	0.0512, 0.1118	0.0731, 0.1724	0.0259, 0.0612	0.0313, 0.0659
Largest diff. peak/hole / e Å^{-3}	0.51/-0.29	0.56/-0.23	0.30/-0.20	0.31/-0.17
Flack parameter			-0.002(19)	0.00(2)
CCDC No.	2466294	2466295	2466296	2466306

Table S10. Bond lengths of d-Trp and h-Trp.

		d-Trp	h-Trp
Atoms		Bond length/Å	
O2	C3	1.265(3)	1.2690(13)
O1	C3	1.251(3)	1.2490(13)
N5	C4	1.499(3)	1.4902(13)
N9	C8	1.373(4)	1.3802(15)
N9	C10	1.383(3)	1.3765(16)
C3	C4	1.528(3)	1.5316(14)
C6	C4	1.541(3)	1.5435(15)
C11	C7	1.441(4)	1.4426(15)
C7	C6	1.497(3)	1.4943(15)
C7	C8	1.369(4)	1.3692(16)
C11	C10	1.419(3)	1.4216(15)
C11	C12	1.407(4)	1.4047(16)
C10	C15	1.391(4)	1.3991(16)
C12	C13	1.381(4)	1.3906(17)
C13	C14	1.407(5)	1.4109(19)
C15	C14	1.388(4)	1.3866(19)

Table S11. Bond angles of d-Trp and h-Trp.

			d-Trp	h-Trp				d-Trp	h-Trp
	Atoms		Bond Angles/°			Atoms		Bond Angles/°	
C8	N9	C10	109.9(2)	109.24(10)	C12	C11	C7	133.7(2)	133.93(11)
N5	C4	C3	109.13(19)	109.49(8)	C12	C11	C10	118.9(2)	119.13(10)
N5	C4	C6	109.72(18)	109.63(8)	C10	C11	C7	107.2(2)	106.91(10)
C3	C4	C6	112.02(19)	111.91(9)	C13	C12	C11	118.3(3)	118.83(11)
O2	C3	C4	118.3(2)	117.58(9)	C12	C13	C14	121.7(3)	120.94(12)
O1	C3	O2	125.8(2)	126.07(10)	C15	C14	C13	121.2(3)	121.57(11)
O1	C3	C4	115.8(2)	116.35(9)	C14	C15	C10	117.0(3)	117.31(11)
C7	C6	C4	113.6(2)	113.61(9)	N9	C10	C11	106.5(3)	107.30(10)
C11	C7	C6	127.4(2)	126.64(10)	N9	C10	C15	130.7(3)	130.44(11)
C8	C7	C6	125.9(2)	126.76(10)	C15	C10	C11	122.8(3)	122.23(11)
C8	C7	C11	106.8(2)	106.59(10)	C7	C8	N9	109.5(2)	109.96(10)

Table S12. Hydrogen bond lengths and angles of d-Trp and h-Trp.

			d-Trp		h-Trp	
**D**	**H**	**A**	**d(D-A)/Å**	**D-H-A/°**	**d(D-A)/Å**	**D-H-A/°**
N5	H5A	O2[1]	2.828(3)	165.9	2.8347(12)	168.2(14)
N5	H5B	O1[2]	2.717(3)	172.5	2.7112(12)	174.1(14)
N5	H5C	O2[3]	2.811(3)	171.2	2.8167(12)	170.7(14)

[1]1-X,1/2+Y,1/2-Z; [2]+X,1+Y,+Z; [3]1-X,1-Y,1-Z

Table S13. Bond lengths of d-TrpCl and h-TrpCl.

		**d-TrpCl**	**h-TrpCl**
Atoms		Bond length/Å	
O2	C3	1.206(2)	1.207(2)
O1	C3	1.325(2)	1.324(2)
N5	C4	1.490(2)	1.490(2)
N9	C8	1.383(2)	1.381(2)
N9	C10	1.378(2)	1.377(2)
C3	C4	1.524(2)	1.522(2)
C4	C6	1.537(2)	1.539(2)
C7	C11	1.446(2)	1.447(2)
C7	C6	1.508(2)	1.506(2)
C7	C8	1.371(2)	1.373(2)
C11	C10	1.419(2)	1.420(2)
C11	C12	1.406(2)	1.405(2)
C10	C15	1.402(2)	1.400(2)
C12	C13	1.386(2)	1.387(2)
C13	C14	1.411(3)	1.410(3)
C15	C14	1.391(3)	1.391(2)

Table S14. Bond angles of d-TrpCl and h-TrpCl.

			**d-TrpCl**	**h-TrpCl**				**d-TrpCl**	**h-TrpCl**
Atoms			Bond Angles/°		Atoms			Bond Angles/°	
C8	N9	C10	109.08(15)	109.15(14)	C12	C11	C7	133.53(16)	133.48(14)
N5	C4	C3	108.53(15)	108.55(13)	C12	C11	C10	119.41(16)	119.40(14)
N5	C4	C6	110.98(14)	110.95(12)	C10	C11	C7	107.06(15)	107.12(13)
C3	C4	C6	113.12(15)	113.09(13)	C13	C12	C11	118.30(17)	118.24(15)
O2	C3	C4	123.56(17)	123.61(15)	C12	C13	C14	121.68(18)	121.71(15)
O1	C3	O2	126.11(17)	125.96(15)	C15	C14	C13	121.22(16)	121.21(15)
O1	C3	C4	110.33(16)	110.42(14)	C14	C15	C10	117.06(17)	117.06(15)
C7	C6	C4	114.96(15)	114.70(13)	N9	C10	C11	107.45(15)	107.39(13)
C11	C7	C6	127.52(16)	127.70(14)	N9	C10	C15	130.22(17)	130.23(15)
C8	C7	C6	126.14(15)	126.11(13)	C15	C10	C11	122.33(17)	122.39(15)
C8	C7	C11	106.34(15)	106.19(13)	C7	C8	N9	110.07(16)	110.15(14)

Table S15. Hydrogen bond lengths and angles of d-TrpCl and h-TrpCl.

			d-TrpCl				h-TrpCl			
**D**	**H**	**A**	**d(D-H)/Å**	**d(H-A)/Å**	**d(D-A)/Å**	**D-H-A/°**	**d(D-H)/Å**	**d(H-A)/Å**	**d(D-A)/Å**	**D-H-A/°**
N5	D5A (H5A)	Cl16[1]	0.89(3)	2.34(3)	3.2112(19)	166(2)	0.90(3)	2.33(3)	3.2123(16)	165(2)
N5	D5B (H5B)	Cl16[2]	0.99(3)	2.31(3)	3.1997(19)	149(2)	0.96(3)	2.34(3)	3.2016(17)	149.4(19)
N5	D5C (H5C)	Cl16[3]	0.91(3)	2.29(3)	3.1717(15)	165(2)	0.88(2)	2.31(2)	3.1699(13)	165(2)
O1	D1 (H1)	Cl16	0.92(3)	2.14(3)	3.0354(15)	167(3)	0.88(3)	2.16(3)	3.0363(14)	168(3)

[1] 1+X,1+Y,+Z; [2] 1+X,+Y,+Z; [3] +X,1+Y,+Z; [3] 1-X,1/2+Y,-Z
